# Effect of PVP on the characteristic of modified membranes made from waste PET bottles for humic acid removal

**DOI:** 10.12688/f1000research.11501.2

**Published:** 2017-06-16

**Authors:** Nasrul Arahman, Afrillia Fahrina, Sastika Amalia, Rahmat Sunarya, Sri Mulyati

**Affiliations:** 1 Department of Chemical Engineering, Universitas Syiah Kuala, Banda Aceh, 23111, Indonesia

**Keywords:** polyethylene terephthalate (PET), plastic bottles, membrane, humic acid

## Abstract

*Background: *The aim of the present study was to evaluate the possibility of using recycled polymer (waste polyethylene terephthalate [PET] bottles) as a membrane material. Furthermore, the effect of the addition of a pore-forming agent and preparation conditions was also observed. *Methods: *Porous polymeric membranes were prepared via thermally induced phase separation by dissolving recycled PET in phenol. PET polymer was obtained from waste plastic bottles as a new source of polymeric material. For original PET membrane, the casting solution was prepared by dissolving of 20wt% PET in phenol solution. For PET modified membrane, a 5 wt% of polyvinylpyrrolidone (PVP) was added into polymer solution. The solution was cast onto a glass plate at room temperature followed by evaporation before the solidification process. The membranes formed were characterized in terms of morphology, chemical group, and filtration performance. A humic acid solution was used to identify the permeability and the solute rejection of the membranes. *Results: *The results showed that the recycled PET from waste plastic bottles was applicable to use as a membrane material for a water treatment process. The maximum flux of 97.0 l/m ^2^.hr was obtained from filtration test using PET membrane. The highest rejection of humic acid in a water sample, which reached up to 75.92%, was obtained using the PET/PVP membrane. *Conclusions: *The recycled PET from waste bottles was successfully used to prepare porous membrane. The membrane was modified by the addition of PVP as a membrane modifying agent. SEM analysis confirmed that the original PET membrane has a rough and large pore structure. The addition of PVP improved the pore density with a narrow pore structure. The PET/PVP membrane conditioned with evaporation was the best in humic acid rejection.

## Introduction

Clean water is one of the most vital and essential elements for sustaining human life. This is the reason why the lack of drinking water has become a serious issue for the entire world ^1, 2^. Membrane technology has been applied widely in water and wastewater treatment processes. In water purification, organic material contaminants, such as humic acid and suspended solids, are effectively removed by microfiltration or ultrafiltration membranes ^3^. The advantages of separation using membrane technology are that it is free of chemicals or additives, uses little temperature or at least less energy compared with conventional treatments (i.e. coagulation followed by sand filter), and that it is scalable and hybrid-separated ^4^.

The effectiveness of the ultrafiltration process using a membrane depends on the material and preparation process. Membranes are prepared from organic substances, such as polymers, or inorganic and composite materials. The polymeric materials generally used in membrane fabrication are cellulose acetate (CA), polyethersulfone, polyvinylidene fluoride, and polyethylene terephthalate (PET). PET is commonly used for membrane ultrafiltration in the separation process. Khayet *et al*. used PET membrane grafting with polystyrene for methanol/toluene separation through pervaporation ^5^, while Behary *et al.* conducted bio-separation from surfactant using PET membrane modified with chitosan ^6^. Li *et al.* used cellulose acetate with PET as an additive for the forward osmosis process ^7^. The effect of PET as an additive is that it increases the mechanical properties of the membrane ^7^.

PET is a polymeric material that is generally derived from commercial polymer, which can increase the production costs. However, plastic bottles as drink packaging are composed of PET); therefore, waste plastic bottles can potentially be used as a membrane material ^8^. This new source of polymeric material helps to reduce the waste of plastic bottles and constitutes a green alternative to limit the consumption of polymers. Additionally, the use of PET bottles as polymeric material will reduce the cost of manufacturing membranes.

The synthesis of PET membranes from plastic bottles was investigated previously by Rajesh and Murti ^9^. Their results showed that PET membranes without modification with polyethylene glycol have poor mechanical properties. Another study by Zander *et al.* investigated using PET from waste bottles to fabricate fiber membranes via the electrospinning technique. The obtained membrane was used for water filtration to separate latex beads. The study found that about 99% of the beads can be removed from a water sample ^10^. In the water treatment process, the pore size of the membrane has an important role in the rejection of water contaminants. Membranes with a small pore size, but high pore density, is recommended to obtain a stable permeation with high selectivity of water contaminant

In this study, the PET membrane from plastic bottles was modified by addition of PVP to enhance its pore size and pore density. The addition of PVP as a pore forming agent and evaporation on the casting film may affect the quality of the resulting membrane. This ultrafiltration PET membrane was then investigated for humic acid removal, and its characterization was performed using the water permeability test, scanning electron microscopy (SEM), Fourier transform infrared spectroscopy (FTIR), and ultraviolet-visible (UV-Vis) analysis.

## Methods

### Materials

PET was derived from waste plastic bottles. Phenol was used as a solvent (KGaA Merck, Germany). PVP (40,000 Da) was purchased from Sigma-Aldrich Co. Ltd (USA). Humic acid (HA) powder was also obtained from Sigma-Aldrich. The HA solution was synthesized by dissolving HA powder in 1 L of distilled water.

### Preparation of the PET membrane

The membrane was prepared via thermally induced phase separation (TIPS). Firstly, phenol, as a solvent, was heated at 50°C until the liquid phase was reached. Fragments of PET bottles were dissolved in molten phenol at 100°C and stirred using a magnetic stirrer for 6 hours until homogeneous. In order to improve the performance of the membrane, 5wt of PVP was added to the solution. Four types of membranes were composed: the composition and the condition of each dope solution are summarized in Table 1. A minimum of four membranes were made of each type, and three membranes of each type were chosen for the filtration experiments (below).

**Table 1.** The composition of dope solution.

Membrane code	Polymer composition (wt%)			Dope condition	Casting temperature (°C)
	PET	PVP	Phenol		
PET-1	20	0	82	Without evaporation	100
PET-2	20	0	82	With evaporation	100
PET-3	20	5	77	Without evaporation	100
PET-4	20	5	77	With evaporation	100

After obtaining a homogeneous solution, the dope temperature was maintained at 100°C without any stirring to remove air bubbles. The homogeneous solutions were cast uniformly onto a glass plate using a Baker applicator (YBA-3, Yoshimitsu, Japan) at room temperature. The thickness of the membrane was set at 700 µm. The casting film was left in the air for 7 minutes to evaporate the solvent. The glass plate was then dipped into a coagulation bath containing water-propanol 1:12 as a non-solvent. Another casting film, for no evaporation treatment, was directly immersed into non-solvent. The glass plate was then dipped into a coagulation bath containing water-propanol 1:12 as a non-solvent. The membrane sheets formed were washed and stored in distilled water for 1 day to remove any residual solvent.

### Membrane morphology

The morphologies of the membrane surface and cross-section were analyzed using a scanning electron microscope (model, JSM 6360LA; JEOL Ltd., Japan). The dried sheets of membrane were gold sputtered for producing electric conductivity. Photomicrographs of PET membranes were viewed in vacuum condition at 5 kV. The magnification image was taken at 10,000 x for the surface and 700 x for the cross-section.

### FTIR spectra

Functional groups of the membrane were analyzed using a Shimadzu FTIR-8400 spectrometer (Japan). A wavelength of 4000–400 cm ^−1^ was used, and the chemical groups of the membranes were identified by their peaks using IR solution 1.50 software (Shimadzu).

### Filtration performance

The ultrafiltration test was conducted using dead-end filtration and pressurized with nitrogen gas. The filtration area of the membranes was 15.2 cm ^2^. The operating condition was set at 1 bar transmembrane pressure and room temperature. Filtration was carried out for 30 minutes, and the permeate was collected three times every 10 minutes. Three repeats for each membrane type was performed. The filtration experiment was evaluated in term of flux and rejection of HA solution. Flux is the total volume of permeate pass through the membrane in a determined filtration period calculated by Equation (1). Rejection is the amount of HA particle rejected by membrane, as analyzed by Equation (2).

$$\text{Flux}=\frac{V}{A.t}\;(1)$$

In which:         V = Volume of permeate (L)

                       A = Membrane surface area (m ^2^)

                       t = Filtration period (hr)

The model for the ultrafiltration test in this study was HA solution at 10 mg/L concentration. The solution was prepared by dissolving HA powder in 1 L distilled water. The rejection value of the membrane in HA filtration was calculated as follows ^11, 12^:

$$R\%=\left(1–\frac{C_{p}}{C_{f}}\right)\times 100\;(2)$$

In which:         R = rejection (%)

                      C _f_ = HA concentration in feed

                      C _p_ = HA concentration in permeate

**Figure 1.**
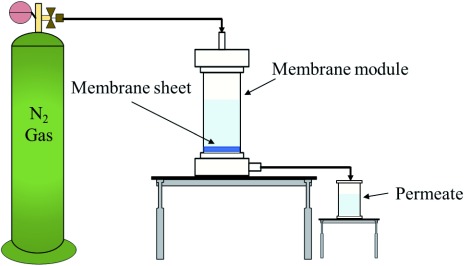
Experimental set up using PET membrane.

The HA concentration in the feed and permeate solution were measured using a UV-Vis spectrometer (model UV-1700; Shimadzu) at 490 nm wavelength. The HA filtration schematic using a PET membrane can be seen in Figure 1.

## Results and discussion

### Membrane morphology

**Figure 2.**
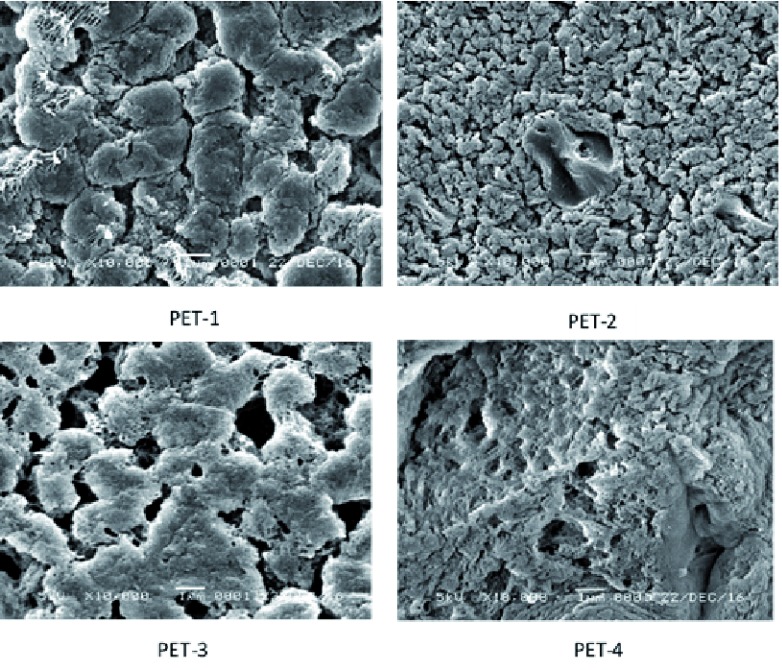
Scanning electron microscopy images of the surface of PET membranes. PET-1, no added PVP or evaporation; PET-2, no added PVP and 7 minutes of evaporation; PET-3, added PVP and no evaporation; added PET-4, PVP and 7 minutes of evaporation.

**Figure 3.**
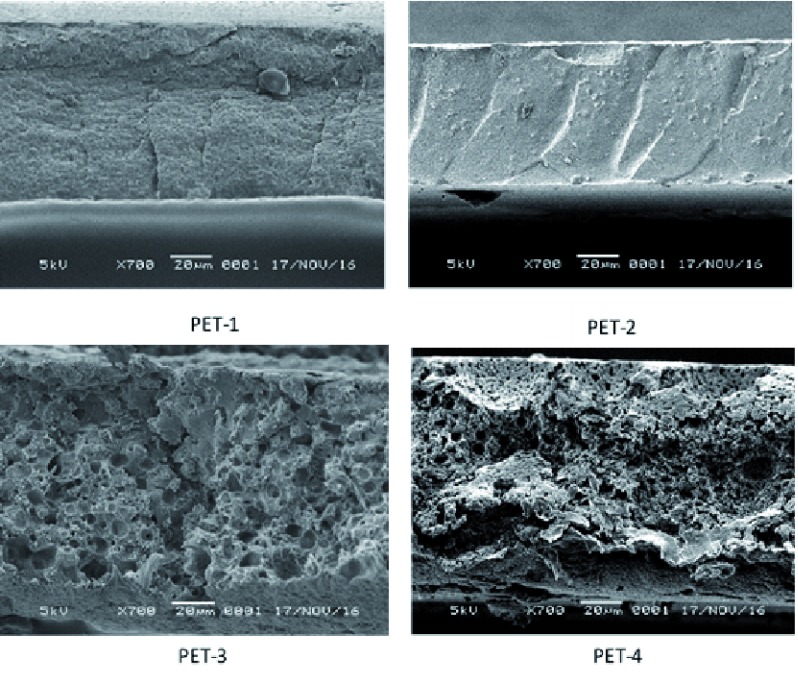
Scanning electron microscopy images of the cross-section of PET membranes. PET-1, no added PVP or evaporation; PET-2, no added PVP and 7 minutes of evaporation; PET-3, added PVP and no evaporation; added PET-4, PVP and 7 minutes of evaporation.

The PET membrane was prepared via TIPS. The constructed membranes were categorized as asymmetric ultrafiltration membrane. The top surface and cross-sectional images of the membranes are shown in Figure 2 and Figure 3, respectively. Figure 2 shows the changes in membrane morphology with the addition of PVP and evaporation on casting film. PET-2 and PET-4 were evaporated for 7 minutes before being immersed in a coagulation bath. The membranes formed showed a decrease in the membrane porosity. A high evaporation temperature (100°C) for 7 minutes increased the exchange rate of solvent from the surface of the casting film. This led to a higher polymer concentration near the top surface, and, therefore, the phase exchange of solvent and nonsolvent in the coagulation bath became lower. This is called delay demixing, which causes the membrane to have a less porous structure ^13^.

The cross-sectional image in Figure 3 shows the asymmetric structure of the sub-layer membrane. The evaporated membranes had a thicker, dense top layer due to the delay demixing. In the sub-layer of the modified membrane, the porous structure changed with the addition of PVP. The effect of PVP in the casting solution caused the formation of pores and sponge structure in the sub-layer ^9^. In the membrane modification process, enhancing the pore density with uniform pore size is essential. A sponge structure, like the one formed in the modified membrane, affects the filtration quality and mechanical properties of the membrane ^2^.

### Membrane functional groups

**Figure 4.**
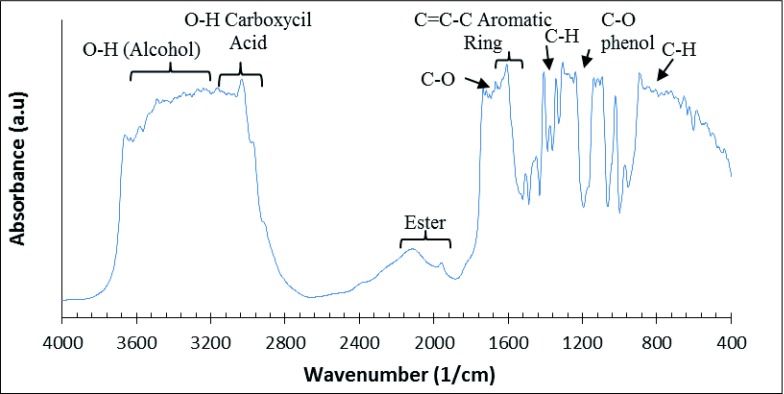
FTIR spectrum of PET original membrane (no added PVP and no evaporation).

**Figure 5.**
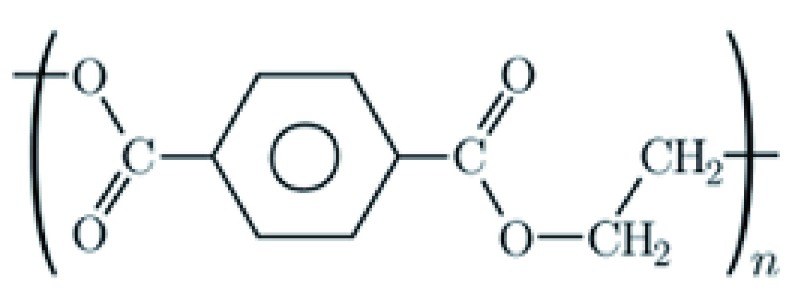
Molecular structure of polyethylene terephthalate (PET) ^16^.

The FTIR spectrum was analyzed to determine the changes of the chemical groups on the membrane surface ^3^. Regarding polymer composition in this research, FTIR analysis was carried out for PET-1 and PET-3 membranes only. Polymer composition of PET-2 and PET-4 membranes was similar with PET-1 and PET-3, respectively; the IR spectra of PET-2 and PET-4 are equal to the IR spectra of PET-1 and PET-3, respectively. Figure 4 shows the FTIR spectrum of the PET-1 membrane. A peak of 3630-3300 cm ^-1^ indicated the presence of the alcohol functional group (OH). In the range of 3200-3000 cm ^-1^ and 1700 cm ^-1^, bands of OH and CO were derived from the carboxylic acid functional group. An aromatic functional group of C = CC band was located at 1650-1600 cm ^-1^. At 1400 cm ^-1^ and 860 cm ^-1^, a C-H band of alkanes and aromatics were observed. A very weak peak at 1250 cm ^-1^ indicated a C-O band of the phenol functional group; phenol is composed of the aromatic ring and OH groups ^14^. The identification of the membrane chemical groups is presented in Table 2. The chemical structure of PET, composed of several chemical groups, is shown in Figure 5. According to the data shown in Table 2, the membrane was composed of PET material.

**Table 2.** Identification of functional groups in PET-1. *Bands of PET polymer cited from reference 15.

Wavelength (cm ^-1^)	Wavelength- based on literature * (cm ^-1^)	Bands	Functional groups
3630-3300	3650-3200	O-H	Alcohol O-H
3200-3000	3300-2500	O-H	Carboxylic Acid
2240-2000	2300-2000	-COO-	Ester
1700	1725-1700	C-O	Carboxylic Acid
1650-1600	1660-1600	C=C-C	Aromatic Ring
1400	1270-1230	C-H	Alkane
1250	1260-1000	C-O	Phenol
860	900-670	C-H	Aromatic

**Figure 6.**
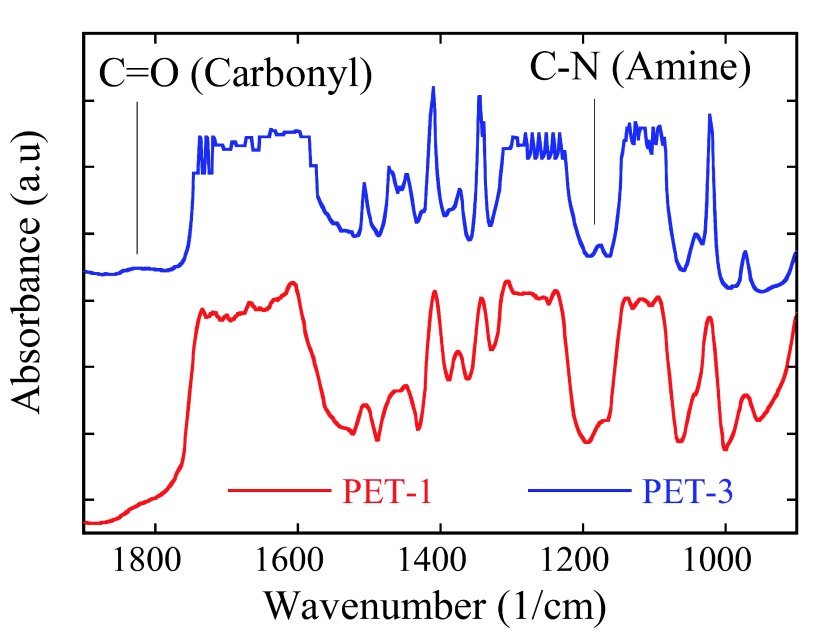
The comparison between the FTIR spectrum of PET-1 and PET-3, a PET/PVP modified membrane. PET-1, no added PVP or evaporation; PET-3, added PVP and no evaporation.

**Figure 7.**
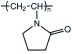
Molecular structure of polyvinyl pyrrolidone (PVP).

The comparison of the FTIR spectrum analysis between PET-1 and modified membranes (PET-3) can be seen in Figure 6. Generally, the FTIR spectrums of PET-1 and modified PET/PVP membranes were similar, because both membranes were made with PET as the basic material. However, a lower peak at 1820 cm ^-1^ and 1180 cm ^-1^ of the modified membrane (PET-3) indicated a carbonyl functional group and CN band. The existence of these bands showed that the membrane was composed of PVP. The molecular structure of PVP is shown in Figure 7.

Raw data for IR spectra (400-4000 (1/cm)) of PET-1 Click here for additional data file. Copyright: © 2017 Arahman N et al. 2017 Data associated with the article are available under the terms of the Creative Commons Zero "No rights reserved" data waiver (CC0 1.0 Public domain dedication).

Raw data for comparison of IR spectra (500-2254 (1/cm)) of PET-1 and PET-3 Click here for additional data file. Copyright: © 2017 Arahman N et al. 2017 Data associated with the article are available under the terms of the Creative Commons Zero "No rights reserved" data waiver (CC0 1.0 Public domain dedication).

### Filtration performance

**Figure 8.**
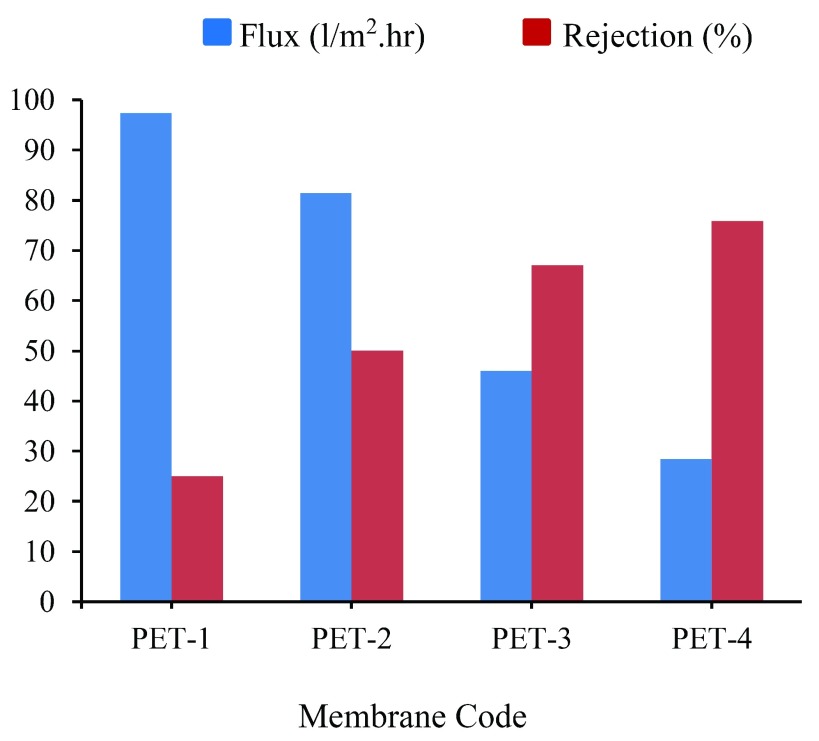
Filtration performance of PET membrane by using humic acid solution. PET-1, no added PVP or evaporation; PET-2, no added PVP and 7 minutes of evaporation; PET-3, added PVP and no evaporation; added PET-4, PVP and 7 minutes of evaporation. Three repeats of each membrane type were performed.

Water filtration is related to membrane characteristics, such as hydrophilicity and pore size. Furthermore, the addition of membrane modifying agent and the evaporation process also affects water permeability ^13^. In this study, a feed solution of humic acid (HA) was tested at 10 mg/L. The concentration of HA solution in the feed and permeate were measured using a UV-Vis spectrometer. The comparison of the original and modified PET membranes in the HA flux and rejection is given in Figure 8. According to Figure 8, the PET/PVP modified membrane with evaporation (PET-4) had the highest HA rejection of up to 76%, followed by PET-3, PET-2, and PET-1 which are 67.3, 50.07, and 25.02%, respectively. The PET original membrane (PET-1) had the maximum flux of up to 97.0 l/m ^2^.hr.

The differences in HA rejection in Figure 8 show the influence of PVP as an additive material and the evaporation time on the performance of the membrane. Figure 8 also shows the flux of the HA filtration. The membrane with smaller and uniform pores (PET-4) was better in HA rejection, but produced less permeates (the addition of PVP in the membrane solution increased the total concentration of the polymer and led the membrane to have smaller pores). Additionally, PVP improved the hydrophilic nature of the membrane surface. This prevented the hydrophobic HA molecules from getting closer to the membrane surface ^3^. Therefore, the PET/PVP- modified membrane followed by evaporation (PET-4) was the best at HA rejection compared to the original PET membrane without evaporation (PET-1), or original PET membrane with evaporation process (PET-2).

Raw data for flux and rejection of humic acid Click here for additional data file. Copyright: © 2017 Arahman N et al. 2017 Data associated with the article are available under the terms of the Creative Commons Zero "No rights reserved" data waiver (CC0 1.0 Public domain dedication).

## Conclusions

Membranes with pore structure were successfully fabricated using recycled PET from waste plastic bottles. The characteristics and performance of these membranes were affected by the membrane preparation conditions. In this study, PET membranes were modified by the addition of additives (PVP) and conditioned using evaporation during solidification. Based on the results, it can be concluded that the presence of PVP in polymer system has an effect on the pore structure and flux of PET membrane. The original PET membrane (PET-1; no PVP or evaporation) had a rough pore structure, which resulted in low solute rejection. The addition of PVP improved pore density with a narrow pore structure, and using a high temperature of evaporation resulted in a membrane surface with smaller pores. Consequently, a PET/PVP membrane conditioned with evaporation (PET-4) was most efficient in humic acid rejection. In general the membranes were suitable for use in a water treatment process. Modifying agents of PET membranes should be further developed to enhance the performance of PET membranes, especially for ultrafiltration process.

## Data availability

The data referenced by this article are under copyright with the following copyright statement: Copyright: © 2017 Arahman N et al.

Data associated with the article are available under the terms of the Creative Commons Zero "No rights reserved" data waiver (CC0 1.0 Public domain dedication). 

Dataset 1: Raw data for IR spectra (400-4000 (1/cm)) of PET-1. doi, 10.5256/f1000research.11501.d160675 ^17^

Dataset 2: Raw data for comparison of IR spectra (500-2254 (1/cm)) of PET-1 and PET-3. doi, 10.5256/f1000research.11501.d160677 ^18^

Dataset 3: Raw data for flux and rejection of humic acid. doi, 10.5256/f1000research.11501.d160679 ^19^
