# Supplementary material for: Effect of PVP on the characteristic of modified membranes made from waste PET bottles for humic acid removal
Source: F1000Res. 2017 Jun 16;6:668. Originally published 2017 May 12. [Version 2] doi: 10.12688/f1000research.11501.2 (PMC5482332; doi:10.12688/f1000research.11501.2)
Supplement: Peer review history

# Peer review: Referee response for version 2

**DOI:** 10.5256/f1000research.12867.r23547

**Published:** 2017-06-19

**Authors:** Zuchra Helwani

**Affiliations:** 1 Department of Chemical Engineering, University of Riau, Pekanbaru, Indonesia

I agree with all revisions

I have read this submission. I believe that I have an appropriate level of expertise to confirm that it is of an acceptable scientific standard.

# Peer review: Referee response for version 2

**DOI:** 10.5256/f1000research.12867.r23546

**Published:** 2017-06-19

**Authors:** Muhammad Roil Bilad

**Affiliations:** 1 Department of Chemical Engineering, Universiti Teknologi Petronas, Bandar Seri Iskandar, Malaysia

In my opinion, authors have address all of my comments properly. I would like to recommend the indexing of the manuscript.

I have read this submission. I believe that I have an appropriate level of expertise to confirm that it is of an acceptable scientific standard.

# Peer review: Referee response for version 1

**DOI:** 10.5256/f1000research.12421.r23115

**Published:** 2017-06-05

**Authors:** Zuchra Helwani

**Affiliations:** 1 Department of Chemical Engineering, University of Riau, Pekanbaru, Indonesia

In the discussion about filtration performance (rejection and flux), give more reasons regarding the best performance of PET-4. Make sure that these reasons are related to the characteristic of the membranes.  The statistical analysis and its interpretation are only partly appropriate - was the filtration performance conducted on different conditions, like pressure and the temperature of the process?

I have read this submission. I believe that I have an appropriate level of expertise to confirm that it is of an acceptable scientific standard, however I have significant reservations, as outlined above.

# Response

**Published:** 2017-06-14

**Authors:** Nasrul Arahman

**Affiliations:** Syiah Kuala Uiversity, Indonesia

According to the SEM image in Figure 2, PET-4 has densest and smallest pore than other membranes. The pore can retain humid acid molecules which has larger pore than the membrane. So, in filtration test, PET-4 has the highest rejection of humid acid. Different pressures were conducted in preliminary permeation test. In case of PET-4 membrane, a maximum 3 atm pressure is applied for permeation test. On the other hand, PET membrane without addition of PVP (PET-1, and PET-3), the permeation test only capable at 1 atm of applied pressure. The addition of PVP brought about the enhance of membrane strength so that it is appropriate to conduct the filtration process at pressure of 2 or 3 atm. This is the reason the filtration performance test was carried out at constant pressure at 1 bar for all membrane. Addition of PVP into polymer solution also influence to increase the hydrophilicity of the membrane. Good hydrophilicity of membrane surface can prevent HA molecules attach to the membrane surface. So, we concluded that PET-4 is the best membrane in this study.

# Peer review: Referee response for version 1

**DOI:** 10.5256/f1000research.12421.r22681

**Published:** 2017-05-22

**Authors:** Muhammad Roil Bilad

**Affiliations:** 1 Department of Chemical Engineering, Universiti Teknologi Petronas, Bandar Seri Iskandar, Malaysia

This study assesses the possibility of using recycled polyethylene terephthalate from used bottle waste as a membrane material. A topic that highly relevant in waste-to-value perspective. The fact that the study was a success make this report worthy of publication. Moreover, the approach of using recycled PET as membrane material is rather new with only few references available. I believe that by addressing comments below, the quality of the manuscript will be improved.

 Major comment:

 When discussing the membrane formation mechanism, the authors mentioned the roles of evaporation time. However, by looking into the properties of phenol (melting point of 40.5 °C and boiling point of 181.7 °C), the rate of phenol evaporation is too low. As seen in Figure 4, phenol in the presence of residue in the membrane matrices is obvious. Another aspect to be discussed is the effect of falling temperature on the solution (I assumed that the casting was done at room temperature) as well as inhibition of water from humid air. Those phenomena occur simultaneously and contribute to the formation of membrane structure.

 The authors are expected to address minor comments below and fit their answer into the revised manuscript.

- Add information about the permeability in the abstract.
- Please revise typo error far description of parameters in Eq. 2: C _p _and C _f_ instead of Cp and Cf.
- What is the solubility of phenol in a mixture of 1:12 of water:propanol. Is there any preliminary study on selecting this ratio? What will be the impact of non-solvent composition on the produced PET membrane?
- Figure 8: A simpler scatter plot will be more informative rather than overlapping 3D plots in the current version of the manuscript. Also, information on the testing pressure should be included in the caption.

I have read this submission. I believe that I have an appropriate level of expertise to confirm that it is of an acceptable scientific standard.

# Response

**Published:** 2017-06-14

**Authors:** Nasrul Arahman

**Affiliations:** Syiah Kuala Uiversity, Indonesia

Comment 1.

 When discussing the membrane formation mechanism, the authors mentioned the roles of evaporation time. However, by looking into the properties of phenol (melting point of 40.5 °C and boiling point of 181.7 °C), the rate of phenol evaporation is too low. As seen in Figure 4, phenol in the presence of residue in the membrane matrices is obvious. Another aspect to be discussed is the effect of falling temperature on the solution (I assumed that the casting was done at room temperature) as well as inhibition of water from humid air. Those phenomena occur simultaneously and contribute to the formation of membrane structure.

 Answer:

 Thank you for your valuable discussion.

 We agree with your opinion. Evaporation treatment is one the affected parameter to the membrane morphology. Another parameter as you pointed was also affected to the membrane formation. The changing of solution temperature suddenly from hot plate condition to glass plate at room temperature may affect the membrane formation. However, we did not conduct such kind of this investigation in this study.

 Comment 2.

 Add information about the permeability in the abstract (revised manuscript).

 Answer:

 Flux information added in the abstract

 Comment 3.

 Please revise typo error far description of parameters in Eq. 2: C _p_ and C _f_ instead of Cp and Cf.

 Answer:

 Type error for description of parameter in Equation 2 already revised (revised manuscript).

 Comment 4. 

 What is the solubility of phenol in a mixture of 1:12 of water : propanol. Is there any preliminary study on selecting this ratio? What will be the impact of non-solvent composition on the produced PET membrane?

 Answer:

 The composition of phenol-propanol (1:12) as non-solvent was determined by conducting several solidification process using a different concentration of water-propanol. The mixture was tested at ratio 1:0; 1:1; 1:5; 1:7; 1:9; and 1:12. The result shows that non-solvent without propanol produced a rigid and kinked membrane with many big holes on the surfaces. The addition of propanol helps to smoothen the surface. But, too much propanol caused mechanical properties of membrane too fragile. So based on the preliminary study, the mixture of 1:12 of water-propanol was the best non-solvent composition to produced PET membrane with good mechanical properties and pore performance.

 The solubility of phenol based on material safety data sheet from sciencelab.com : Easily soluble in methanol, diethyl ether. Soluble in cold water, acetone. Solubility in water: 1g/15 ml water. Soluble in benzene. Very soluble in alcohol, chloroform, glycerol, petroleum, carbon disulfide, volatile and fixed oils, aqueous alkali hydroxides, carbon tetrachloride, acetic acid, liquid sulfur dioxide. Almost insoluble in petroleum ether. Miscible in acetone. Sparingly soluble in mineral oil.  So, phenol has good solubility in a mixture of water-propanol.

 Comment 5.

 Figure 8: A simpler scatter plot will be more informative rather than overlapping 3D plots in the current version of the manuscript. Also, information on the testing pressure should be included in the caption.

 Answer:

 New figure prepared and replaced for Figure 8 (revised manuscript).
